# 3D Printing Wood–PLA Composites: The Impact of Wood Particle Size

**DOI:** 10.3390/polym17091165

**Published:** 2025-04-25

**Authors:** Wojciech Jasiński, Karol Szymanowski, Barbara Nasiłowska, Marek Barlak, Izabela Betlej, Artur Prokopiuk, Piotr Borysiuk

**Affiliations:** 1 Institute of Wood Sciences and Furniture, Warsaw University of Life Sciences-SGGW, 159 Nowoursynowska St., 02-776 Warsaw, Poland; karol_szymanowski@sggw.edu.pl (K.S.); izabela_betlej@sggw.edu.pl (I.B.); 2 Institute of Optoelectronics, Military University of Technology, gen. S. Kaliskiego 2 St., 00-908 Warsaw, Poland; barbara.nasilowska@wat.edu.pl (B.N.); artur.prokopiuk@wat.edu.pl (A.P.); 3 Ion Beam Technology Division, Material Physics Department, National Centre for Nuclear Research Świerk, 7 Sołtana St., 05-400 Otwock, Poland; marek.barlak@ncbj.gov.pl

**Keywords:** printability, tensile properties, SEM, FDM, nozzle size

## Abstract

Particle size of wood fillers used in FDM 3D printing filaments is a topic not commonly discussed in the literature. Research on traditional wood–polymer composites (WPCs) suggests that bigger particles improve the composite’s tensile properties. Is that the case at the 3D printing scale? Five variants of composites were prepared using recycled PLA and sawdust, differentiated by filler particle size (<0.2 mm, 0.2 mm–0.4 mm, 0.4 mm–0.6 mm, 0.6 mm–0.8 mm, 0.8 mm–1 mm). Current draw during extrusion, as well as tensile strength and tensile modulus, were tested. Test results of tensile strength, ranging from 9.21 MPa to 14.28 MPa, and tensile modulus, ranging from 802 MPa to 1014 MPa, have shown no clear correlation between wood particle size and tensile properties of the composites at the 3D printing scale. A clear increase in forces needed to extrude composites containing larger particles of wood was discovered, as well as the inability to extrude composites filled with the biggest tested particle size. To further explore this topic, SEM/EDS imaging of the tested composites was performed. Based on the test results, wood particle sizes ranging from one-fifth to one-half of the nozzle size are recommended for use as fillers in wood–PLA composites intended for 3D printing.

## 1. Introduction

Wood–polymer composites (WPCs) are a branch of vastly diverse materials created mainly using polymers (matrices) and wood (fillers). Usually polymers used in manufacturing of WPCs are petroleum based, although use of biopolymers as matrices is becoming increasingly popular [1,2,3]. Properties of the most widely researched biopolymer, polylactic acid (PLA), are similar to those of polyethylene (PE) and polystyrene (PS). However, PLA exhibits a lower melting point and heat deflection temperature [4,5,6]. Lower processing temperatures as well as low thermal expansion cause PLA to be the most popular polymer used in fused deposition modeling (FDM) 3D printing.

Wood particles used as fillers in WPCs are often by-products of the wood industry, usually in forms of wood fibers or sawdust [7]. Previous studies widely describe the impact of different types and forms of lignocellulosic fillers on the properties of WPCs. No overall limitations exist in terms of wood types used as fillers; however, the higher slenderness of coniferous species’ structural elements renders them more suitable than deciduous species [8,9]. Compatibility of particular fillers and matrices is a key factor regarding WPC properties, as weak adhesion between those, resulting in discontinuities between the polymer and wood particles, leads to degradation of mechanical performance [10]. Gozdecki et al. [11] and Landon et al. [12] report an increase in the tensile properties of WPCs filled with larger particle sizes, although Włodarczyk-Fligier et al. [13] and Ratanawilai et al. [14] describe larger particles as negatively impacting the mechanical properties of WPCs. These contradictory data suggest that the impact of wood particle size on the mechanical properties of WPCs is either non-linear or varied between different WPC compositions and requires further testing for composites intended for used in FDM 3D printing.

Most available publications explore traditional extruded, pressed, or injection-molded WPCs. Three-dimensional printing, and especially 3D printing with WPCs, is a relatively modern technology that lacks scientific coverage. While most phenomena regarding traditional composites may be assumed to also be true for 3D printed composites, the crucial difference between standard extruded composites and 3D printing composites is the nozzle size. Three-dimensional printers are usually equipped with nozzles ranging from 0.4 mm to 0.6 mm in diameter, drastically limiting the size of particles that can be used in this technology. Less common, yet still widely used and commercially available, nozzles range from 0.2 mm to 1.8 mm [15], and rarely 2.0 mm, in diameter. These values still illustrate a substantial difference between 3D-printed and traditionally extruded composites. A significant amount of research on this topic has been performed by filament manufacturers; however, results of these studies are rarely published.

Testing of mechanical, and especially tensile properties of 3D-printed WPCs or 3D-printed filaments in general, presents challenges caused by the specificity of the FDM technology. Due to a lack of standards regarding tests of 3D-printed samples, many authors tried to adapt pre-existing standards, such as ISO-527, or its ASTM equivalent to perform their research. Such adaptations while using 3D-printed samples of the normative “dog bone” shape may introduce skewness of the test results caused by inconsistencies in the printing process and even provide invalid test results due to gaps in the areas of the sample where its thickness transitions between its narrow part and gripping points [16]. Due to the lack of standards and issues in testing 3D-printed samples, filament tensile testing has been proposed by Rodrigues et al. [16] and Sola et al. [17]. This testing involves similar procedures to the standards described above, but uses filament samples instead of 3D-printed samples. This method is reported to provide accurate and consistent results, reliably evaluating properties of the tested materials.

While several studies regarding tensile properties of FDM WPCs have been published, most of those describe either the impact of wood content [18], the impact of printing process parameters, such as printing speed [19] or the impact of wood type [20,21]. Only one publication regarding the impact of particle size on the tensile properties of FDM WPCs was found at the time of this study. Huang et al. [22] explored this subject, concluding that no significant impact of particle size on the tensile properties of 3D-printed WPCs could be found.

The topic of printability of WPCs in FDM technology may be found in the literature, although most of these publications describe printability as a binary possibility of printing with the composite (can/cannot be printed) [23] or, more often, the quality of printed parts [24,25], which may be a result of improper attunement of the 3D printing process and its multiple variables to the tested material, rather than the material’s properties. No studies describing the impact of wood particle size on forces needed to extrude a 3D printing wood–PLA composite were found prior to the research described in this study.

Studies regarding non-filled filaments have described the impact of multiple parameters on the printability of these materials, often concluding their viscosity as the most crucial parameter, with polymer relaxation time a key factor in the post-extrusion solidification process and final quality of the parts [26,27]. These findings however cannot fully be applied to WPCs, which are composed of more than thermoplastic materials.

The growing popularity of 3D printing in both home and production environments paired with gaps in available research, conflicting studies, and the lack of normative references show the need to further explore this subject. The aim of this study is to quantify the impact of particle size on the tensile properties and printability of WPCs intended for 3D printing, while also exploring the limitations caused by the extrusion scale of FDM 3D printing.

## 2. Materials and Methods

**Figure 1:**
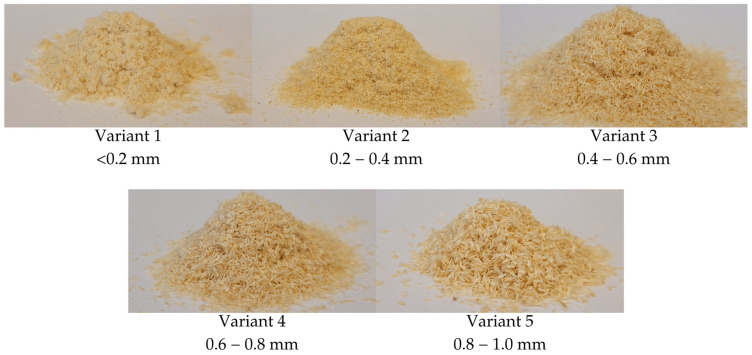
Sizes of wood particles used in this study.

Five variants of wood–polymer composites, differentiated by wood particle size, were manufactured using recycled PLA granulate (Ingeo™ biopolymer 4043D, NatureWorks LLC, Minnetonka, MN, USA) and pine sawdust. Prior to the research described herein the polymer was mixed with pigments in a 99:1 (polymer:pigment) weight ratio and extruded once. Given that the extruded product exceeded dimensional tolerances, the PLA was mechanically ground and recycled. Pine sawdust was mechanically ground and sorted using sieves, as shown in Figure 1. It was then dried to a 5% ± 1% moisture content. The moisture content of the used materials, as presented in Table 1, was measured by calculating the weight difference before and after oven drying to a constant mass. Dried, ground, and sieved sawdust was mixed with PLA in a 10:90 (wood to polymer) weight. The choice of 10% wood content was based on the research by Zeng et al. [28], who described that while tensile properties of WPCs increased with an increase in wood content, the overabundance of wood particles may cause particle agglomeration and improper encapsulation of filler in the matrix. A lower wood content than usually found in commercial 3D printing WPCs was chosen to match the optimum for tensile properties as closely as possible and ensure proper homogenization of the composites. No other additives were used. Variant 0, which contained no wood particles, was also prepared.

**Figure 2:**
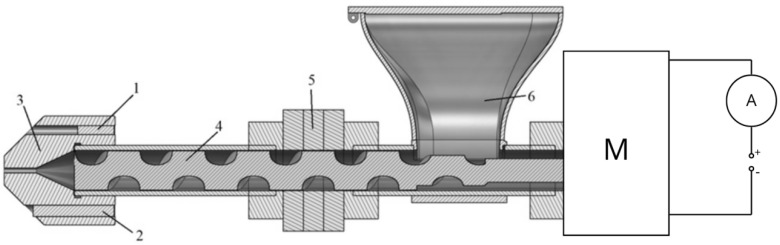
Test setup: 1—thermocouple, 2—heating element, 3—head, 4—screw, 5—radiator, 6—hopper, M—motor, A—ammeter.

The impact of particle size on the printability of WPCs could not be directly tested due to 3D printer’s extruders being usually equipped with stepper motors, which operate with constant torque. Printability tests performed directly using a 3D printer yield binary results (can/cannot be printed) [23] because the torque needed to extrude the composites can either be lower or higher than the torque set in the stepper motor driver. Instead, the tests were performed using an original design filament extruder (Figure 2) equipped with a 24 V DC motor and a single 20 mm diameter screw with a length to diameter ratio (L/D) of 13.5:1. Use of an extruder instead of a standard 3D printer also eliminated the issue of filament buckling between the feeding and melting zones of the 3D printing toolhead, which could have skewed the test results [27]. Previously mixed variants were extruded through a nozzle with a diameter of 1.5 mm with a heating element set at 150 °C ± 3 °C. Although a 1.5 mm diameter nozzle is not typical in FDM 3D printing, nozzles of that size are widely available for most standard 3D printers and allow testing of a wider selection of wood particle sizes. A larger nozzle size also allowed for lower processing temperature, which limited the degradation of both polymer and wood particles during processing. During extrusion, the current drawn by the motor was measured with a 1 Hz polling rate to approximate the torque needed to extrude the composites. Three 10-min runs were recorded for each tested variant, with up to three additional runs in case a run could not be finished. The runs were performed after at least 30 min of the extruder’s operation to ensure constant operating temperatures.

Tensile strength and tensile modulus were tested according to ISO-527 [29] using cylindrical samples with a diameter of 2 mm and a length of 170 mm. Each of these tests involved 15 repetitions. The choice of cylindrical samples was based on the research conducted by Rodrigues et al. [16] and Sola et al. [17], who described the impact of the 3D printing process and its shortcomings on consistency and viability of tensile tests, in which 3D-printed samples were used. A combination of normative procedures and apparatuses (including standard machine grips) and cylindrical samples of the filament extruded during previous tests could eliminate the negative impact of further processing. Samples of this size however could exaggerate the impact of low wood–PLA adhesion and significantly lower the results of the tensile tests. As such, data presented in this study should only be used as a relative comparison between the tested variants’ properties and not as a realistic performance prediction. While the behavior of samples of wood–PLA composites during tensile tests was as expected, several samples of pure PLA (variant 0) exhibited either slipping in the grips or breakage at the edge of the grips. Further samples had to be tested to account for those unviable results. This suggests that in the future, especially for non-filled polymer testing, a different gripping apparatus should be explored. Analysis of variance (ANOVA) tests with post-hoc Tukey tests were used to determine the significance of differences between variants. These tests were performed using STATISTICA 13 (TIBCO Software Inc., Palo Alto, CA, USA) with a confidence level of 0.95.

Two series of scanning electron microscopy (SEM) observations were performed, including before and after the tensile tests. First, observations of the tested composites were performed using a Zeiss EVO^®^ MA10 SEM (Oberkochen, Germany), with two basic type detectors, i.e., a secondary electron detector (marked as SE or SED) and backscattered electron detector (BSE or BSD). The observations were possible only for small value of the beam current (I Probe = 50 pA) because the fast degradation of the material surface was observed for the higher values of the beam current. The investigated samples were covered with an approximately 5 nm gold layer before the observations, using a Q150R ES rotary pumped coating system by Quorum Technologies Ltd. (Laughton, UK), for the purpose of improved poor electrical conductivity. Subsequently, the fracture structure of the composites was imaged using a Quanta 250 FEG SEM (FEI, Hillsboro, OR, USA), equipped with an ETD-BSE backscattering detector (FEI, Hillsboro, OR, USA). The tests were conducted at an accelerating voltage of 10 kV. Before imaging, the composite samples were sputtered with a 6.16 nm thick gold layer. An EM ACE 600 sputtering machine (Leica Microsystems GmbH, Wetzlar, Germany) was used for this purpose. During the sputtering process, the table on which the samples were placed was rotated and tilted at 120°. Discontinuity measurements were performed by analyzing graphic files from an electron microscope. These images were imported into CorelDRAW version 2019 (Corel Corporation, Ottawa, ON, Canada), and then the image dimensions were resized according to the scale obtained from the microscope. The dimensions of the discontinuities were determined using the “Parallel dimension” tool built into the application, which allowed measurement of the actual absolute values, expressed in µm.

**Figure 3:**
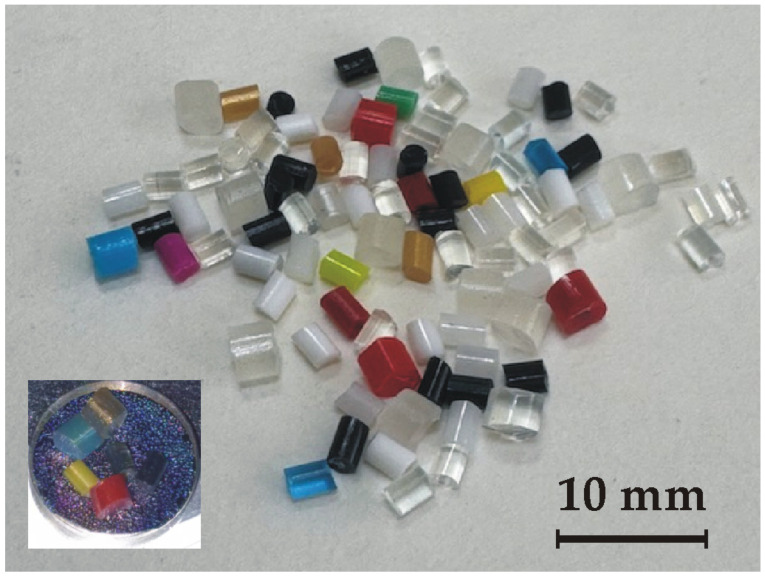
The recycled, initial (non-extruded) PLA material.

The (semi-quantitative) chemical composition of the non-extruded and the extruded PLA was analyzed using an EDX Bruker XFlash Detector 5010 (Bruker Corp., Billerica, MA, USA) energy-dispersive spectroscopy (EDS) system with the dedicated Quantax 200, Esprit 1.9 code. This system is an integral part of the Zeiss EVO^®^ (Oberkochen, Germany) MA10 SEM described above. A fast (5 min/measurement) standardless ZAF method was applied, which accounts for correction coefficients for atomic number Z, absorption A, and fluorescence F. An EDS analysis of the surfaces of the investigated materials was performed for the typical magnifications of ×1000. The investigated area was 269 × 202 μm^2^ for this value. The applied acceleration voltage of the electrons was 20 kV. The electron interaction depth, which was understood as the electron penetration depth, and the radius, which was understood as the electron beam diameter, were in the range of 1.1–1.8 µm and 0.6–1.1 µm, respectively. The investigated samples were covered with an approximately 5 nm gold layer before the observations and the analysis using a Q150R ES rotary pumped coating system by Quorum Technologies Ltd. (Laughton, UK) for the purpose of improving their poor electrical conductivity. The recycled, initial (non-extruded) PLA material is presented in Figure 3. The insert in the lower left corner shows the macrograph illustrating the six selected analyzed samples on the microscopic sample holder.

## 3. Results and Discussion

### 3.1. Printability

**Figure 4:**
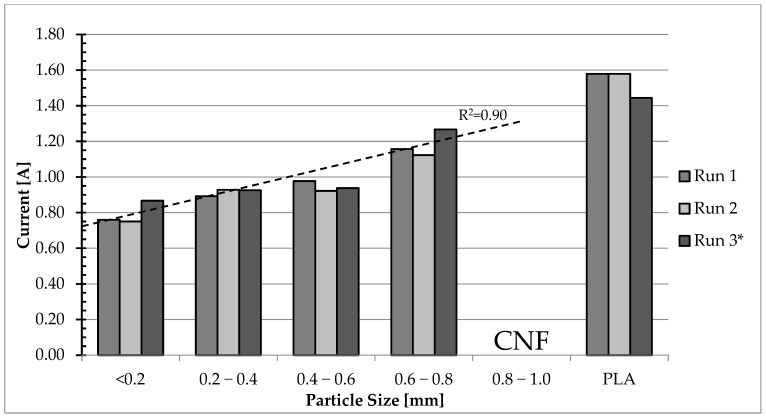
Average current draw for each tested variant; * Run 3 for a particle size of 0.6–0.8 mm could not be finished. An additional run is shown instead. CNF—Runs could not be finished. PLA—Variant 0 with no wood particles.

Average current draw during extrusion (Figure 4) ranged from 0.79 A for the composite filled with the smallest particles (<0.2 mm) to 1.27 A for the composite filled with the second largest particles (0.6–0.8 mm). A significant difference in current draw could be seen between all the tested composite variants and the control group, containing only recycled PLA. The average current draw for variant 0 is nearly twice as high as the average for variant 1. This difference is probably caused by differences in the rheological properties of PLA and wood–PLA composites, namely the lower viscosity of WPCs caused either by the higher content of water, which was contained in the wood particles [30], or the lower density caused by the addition of wood particles. Further rheological testing would however be required to unequivocally explain this observation. A near-linear correlation between particle size and current draw may be observed in the results of these tests, which shows that more force is needed for the extrusion of composites filled with bigger particles. Depending on the 3D printer’s construction, this may cause difficulties with printing these composites and, in certain cases, could prohibit their use in 3D printing.

The third run for variant 4 had to be repeated due to a clog in the nozzle after 7 min of the run. Despite three additional attempts, no runs for variant 5 were completed for the same reason, each failing after up to 3 min of the run. This suggests that particles of sizes larger than half of the nozzle diameter (0.75 mm) are not suitable for use in composites intended for 3D printing.

**Figure 5:**
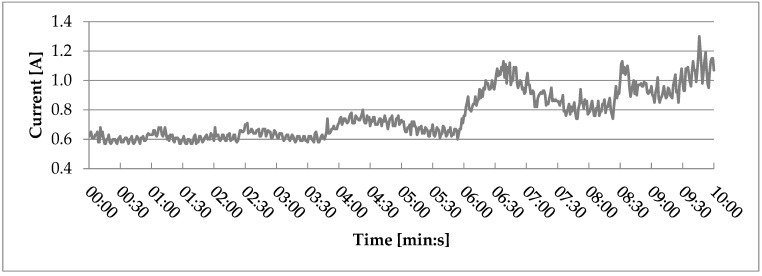
Current drawn during the first test run for variant 1, filled with wood particles sized <0.2 mm.

While variants filled with particles smaller than half of the nozzle diameter did not fully clog the extruder nozzle, partial clogs could be observed for variant 1, filled with the smallest particle size. This partial clogging, and the rise in current draw caused by it, was the most noticeable during run 1 (Figure 5), starting at the 6th minute of the run.

**Figure 6:**
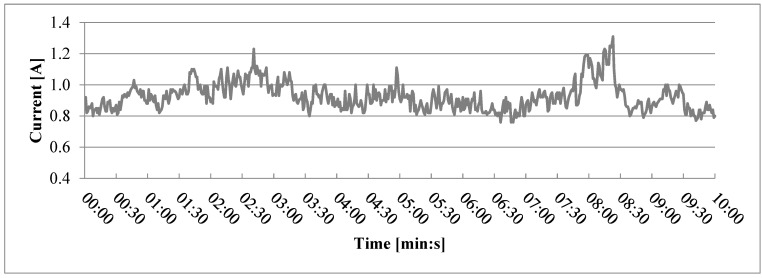
Current drawn during the third test run for variant 2, filled with wood particles sized 0.2 mm–0.4 mm.

**Figure 7:**
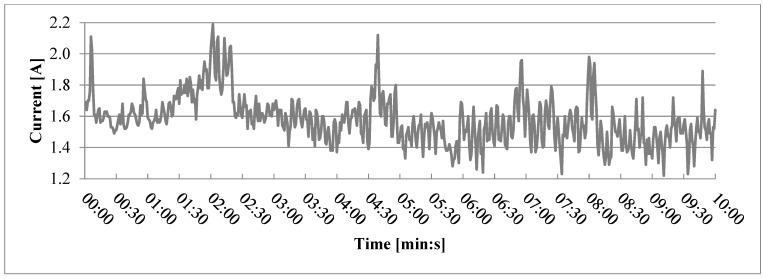
Current drawn during the second run for variant 0 (without wood particles).

Current draw during extrusion of other variants, including variant 2 (Figure 6), was less stable than current draw during extrusion of variant 1, but no clear clogging was noticed. Similarly, even greater instability could be noticed during extrusion of variant 0 containing no wood particles (Figure 7). Based on this, overall current draw instability can be attributed neither to particle size nor the presence of wood particles. Partial clogging issue could be caused by conglomeration of the finest particles due to their high specific surface area [31] and would probably be aggravated at the usual 3D printing scale, which is even smaller than the one shown in this study.

Tests performed by Petchwattana et al. [23] show that, while using a 0.4 mm diameter nozzle, composites filled with particle sizes of 18.5% of nozzle diameter (0.074 mm) could be used on a 3D printer, whereas particle sizes of 31.5% of nozzle diameter (0.125 mm) could not. These data are significantly lower than the tests described in the present study would suggest; however, the authors describe only that the printability test for the larger particle size failed and suggest the reason to be particle agglomeration, without describing details of the failure. Huang et al. [22], using a 0.8 mm diameter nozzle, successfully printed WPCs filled with wood particles up to a size of 26.5% (0.212 mm) of nozzle diameter, with no particles exceeding the range in which 3D printing is possible. Most other reports used even smaller particles and/or smaller particle size to nozzle size ratios. The inability to use composites with lower particle sizes in FDM 3D printing is attributed of one of two reasons—either the forces required to extrude the composite filled with larger particles exceeded the torque supplied by the stepper motor in the 3D printer’s extruder or the ratio of particle size to nozzle size that causes clogging is different for different nozzle sizes. Based on the available data, the former is more likely; however, further testing using a wider selection of nozzle sizes is needed to accurately describe these differences.

The literature describing the impact of wood particle size on rheological properties of WPCs indicates that an increase in particle dimensions causes a decrease in the composite’s viscosity [32]. This fact is in contradiction with the data obtained during printability tests, as lower viscosity should result in a decrease of forces needed to extrude the composite, resulting in a lower current draw during extrusion. This apparent discrepancy suggest that rheological differences between the tested composites play a role secondary to mechanical interactions between the wood particles and nozzle orifice at this scale.

Conglomeration occurring during extrusion of the finest particle filled composites and the increase in force needed for the extrusion of composites filled with larger particles are not the only factors that should be regarded while considering the filler particle size. Comminution of the wood particles to smaller sizes requires significantly higher energy input and should also be considered in the production process of 3D printing WPCs [33].

### 3.2. Tensile Properties

Tensile properties of the tested composites (Table 2) were significantly lower than the data available in the literature [34], which was expected due to non-normative dimensions of the tested samples. The average tensile strength of the tested composites ranged from 9.21 MPa for variant 3 to 14.28 MPa for variant 4. The difference between the aforementioned variants was the only statistically significant difference between WPC variants in this test.

Similarly, tensile modulus differences between individual variants in this test were either minimal or in most cases insignificant. The average tensile modulus of tested composites ranged from 802 MPa for variant 2 to 1014 MPa for variant 3. No relationship between particle size and tensile properties could be found based on these data. This fact is in contrast to information available in the literature for the traditional WPCs, where tensile strength was reported to rise in line with increased particle size [11,12] and the tensile modulus is described as being lower for variants filled with bigger particles [21]. On the other hand, results provided by Huang et al. [22] have shown that particle size at the 3D printing scale has minimal to no impact on the tensile strength and tensile modulus of the composites, which is in line with the results obtained in this study. While hydrolytic degradation of the polymer, caused by the presence of water during extrusion, could impact properties of the tested composites, no correlation between moisture content (Table 1) and tensile properties could be noticed during this study. As such, the possibility of water-induced degradation of PLA should be noted. However, based on the data described above and the data available in the literature [30], its impact on the observed trends is probably insignificant.

Further analysis has shown that the average of the three highest values of tensile strength obtained during this study (Table 3) were significantly higher for variant 4, filled with the largest particles used in this test. This suggests improved tensile strength in line with previous research, although only above a certain particle size. Averages of the three lowest tensile strength values did not differ among the tested variants, implicating similar failure mechanisms between these samples caused either by low wood–polymer adhesion or particle conglomeration at the point of breakage.

These phenomena could be caused by objectively small particle sizes used in this study. This suggests that on the 3D printing scale of extrusion, the impact of particle size on the tensile properties WPCs is minimal. Other factors, such as particles’ shape, wettability, adhesion between the matrix and the filler, and the materials themselves, could impact the tensile properties of the composites to a significantly higher degree.

### 3.3. SEM/EDS Results

**Figure 8:**
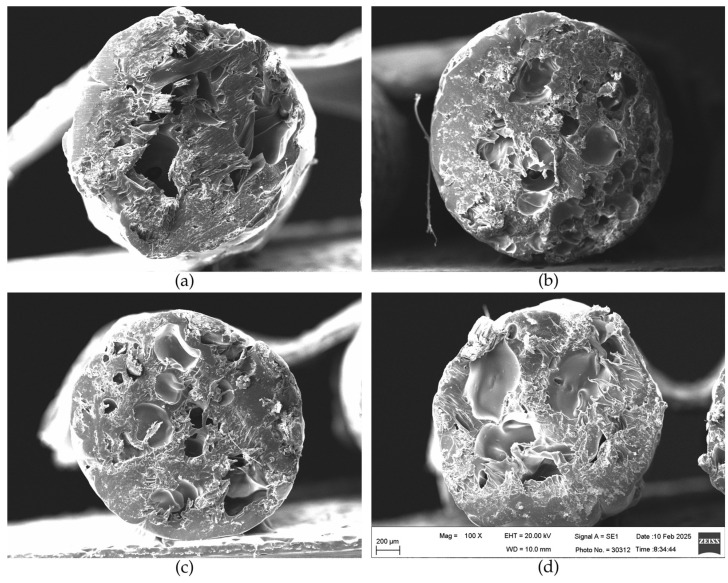
Comparison of SEM images of the tested composites (before the tensile test): (**a**) variant 1; (**b**) variant 2; (**c**) variant 3; (**d**) variant 4.

**Figure 9:**
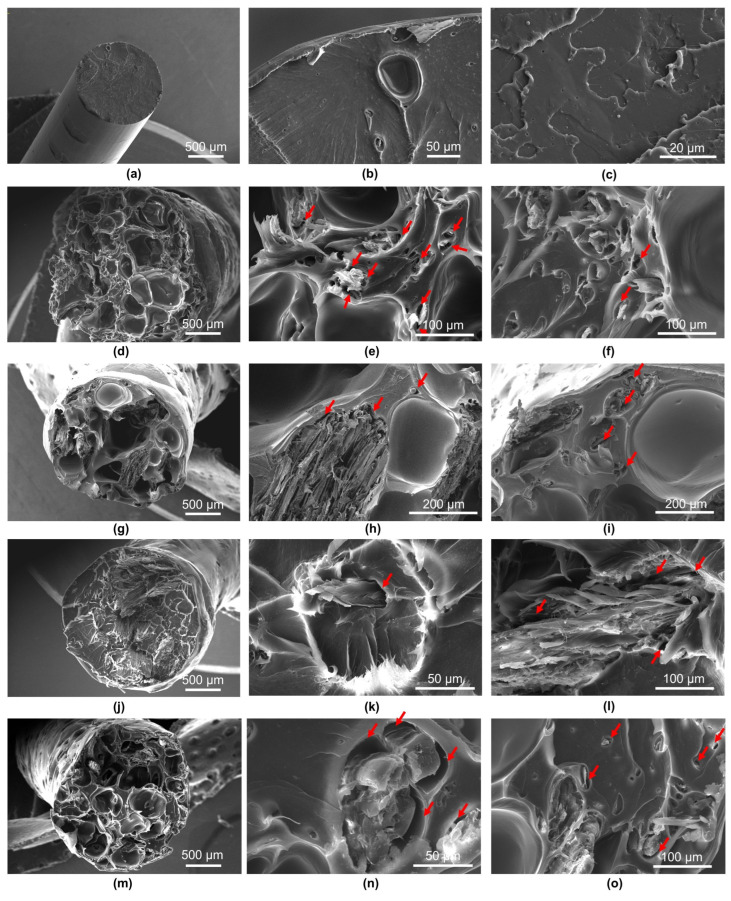
Comparison of SEM images of the tested composites (after the tensile test): (**a**–**c**) variant 0; (**d**–**f**) variant 1; (**g**–**i**) variant 2; (**j**–**l**) variant 3; (**m**–**o**) variant 4; arrows indicate discontinuities between the filler and the matrix.

**Figure 10:**
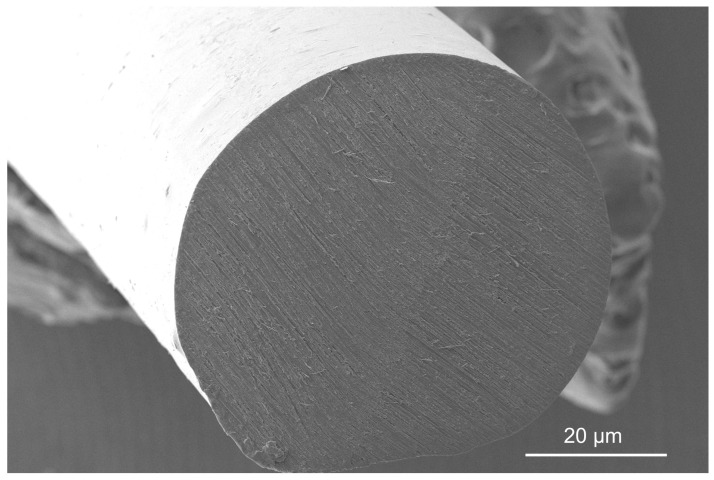
SEM image of variant 0 (before the tensile test).

Figure 8 and Figure 9 show cross-sections of the composites before and after tensile strength tests. The material characteristics on the cross-section were performed for all the research variants, i.e., for samples differing in the size of lignocellulosic particles. SEM images reveal a partially compact structure in the composite’s external part and empty spaces. The presence of empty spaces can undoubtedly be related to the presence of lignocellulosic particles, which are porous materials that accumulate water and air molecules in their capillary spaces, which, as a result of the high temperature during the composite production process, escape from the interior of the wood [18]. This observation can also be confirmed by comparison of the composites’ cross-sections with the cross-sections of variant 0 (Figure 10), containing only recycled PLA. Deaeration of wood particles could be a sufficient method to avoid the lack of continuity of the composite structure. Huang et al. [22] demonstrated that wood particles with more rounded shapes and smoother surfaces would provide better adhesion between the wood and the PLA matrix, thus creating denser and stronger 3D-printed WPC products. Additionally, the same authors suggest that the dual extrusion process can minimize the porosity of the composite.

The cross-sectional morphology after the tearing tests shows that during the elongation of the filament, the voids become more extensive, which is obvious and additionally reveals the lack of compatibility between PLA and lignocellulosic particles (Figure 9d–o). The cross-sections of the composites show structural discontinuities and voids between PLA and wood particles, as indicated by arrows (Figure 9e,f,h,i,k,l,n,o).

Table 4 shows the dimensions of the voids created as a result of the discontinuity of the material between the matrix polymer and the wood particles. The dimensions of the voids ranged from 9.4 to over 40 µm. At the same time, taking into account the size of the wood particles in the individual variants of the filament, it seems that the size of the particles had no effect on the size of the voids between the composite components.

Table 5 presents the average weight percentages of carbon and oxygen in the extruded and non-extruded PLA, after the subtraction of the weight percentage of gold from the deposited layer. It should be remembered that since the EDS analysis does not take into account elements lighter than boron, therefore, based on the PLA (C_3_H_4_O_2_)_n_ formula, the shares of both elements are overestimated by about 5%. Additionally, the standard deviation values were presented. In the both cases, the determined weight percentages of carbon and oxygen are different in the comparison with the values calculated from the mentioned above PLA formula (about 52.97 wt.% of C and 47.03 wt.% of O). Most likely, this is related to the relatively low electron beam penetration depth and oxygen absorbed on the surface of the investigated samples. Additionally, both elements were determined via net-count ratios. The differences of the weight percentages of carbon and oxygen in non-extruded and extruded PLA materials are about 1.14% and −0.75%, respectively. Although the differences are negligible, it should be noted that the material after extrusion is more susceptible to damage associated with electron beam interaction.

These data suggest that no significant degradation of the polymer occurred during extrusion. The main mechanism of both thermal and hydrolytic degradation of PLA is hydrolysis of the ester bonds in the polymer chain, which should result in additional oxygen atoms being present in the degraded product, changing the carbon to oxygen ratios [35]. This result is probably caused by short residence time of the material, i.e., the time that the polymer is being processed in the extruder [36] and lower than usual processing temperatures used in this study (150 °C ± 3 °C), as significant degradation of PLA is reported to occur above a temperature of 200 °C [37]. Further testing, including measurements of molecular mass of the polymer before and after extrusion, would however be needed to explicitly determine the level of degradation.

## 4. Conclusions

No clear correlation between filler particle size and tensile properties of WPCs could be noticed at the 3D printing scale. A near-linear correlation of filler particle size to forces required during composites extrusion was found during this study, which, in conjunction with the biggest tested particle sizes causing nozzle clogs, suggests that particle size should be limited to less than one-half of the nozzle diameter. Partial clogging occurring during extrusion of the composites filled with the finest wood particles, as well as high energy requirements of comminuting wood particles to these sizes, suggest that a lower limit of filler particle size exists. Based on the data obtained during this study, wood particle sizes ranging from one-fifth to one-half of the nozzle diameter are recommended to be used as fillers in WPCs intended for 3D printing with no significant impact on the tensile properties of these composites. This recommendation however is based solely on data described in this study, in which a larger, 1.5 mm diameter nozzle was used. It may not be applicable to all WPC formulations and printing setups. Further research including rheological testing and different nozzle sizes is required to confirm it, as these ranges may differ for different nozzle sizes.

## Figures and Tables

**Table 1:** Moisture content of the materials used in this study.

Material	Particle Size [mm]	Moisture Content
PLA	-	0.54%
WP	<0.2	4.45%
WP	0.2–0.4	4.78%
WP	0.4–0.6	5.58%
WP	0.6–0.8	5.43%
WP	0.8–1.0	5.82%

PLA—Polylactic Acid, WP—Wood particles.

**Table 2:** Average tensile strength and tensile modulus for the tested materials.

Variant	Particle Size [mm]	Tensile Strength [MPa]	Tensile Modulus [MPa]
0	-	33.43 ^A^	1580 ^a^
1	<0.2	12.53 ^BC^	926 ^bc^
2	0.2–0.4	11.32 ^BC^	802 ^bc^
3	0.4–0.6	9.21 ^B^	1014 ^b^
4	0.6–0.8	14.28 ^C^	960 ^c^
5	0.8–1.0	-	-

^A,B,C,a,b,c^—Homogenous groups.

**Table 3:** Average of the three highest and three lowest values of tensile strength for the tested materials.

Variant	Particle Size [mm]	Tensile Strength [MPa]	
		Highest	Lowest
0	-	37.92 ^A^	27.72 ^a^
1	<0.2	16.51 ^B^	6.78 ^b^
2	0.2–0.4	15.36 ^B^	6.05 ^b^
3	0.4–0.6	15.08 ^B^	5.75 ^b^
4	0.6–0.8	19.61 ^C^	6.73 ^b^
5	0.8–1.0	-	-

^A,B,C,a,b^—Homogenous groups.

**Table 4:** Linear measurements of surfaces containing material discontinuity.

		N Total	Mean [µm]	Standard Deviation	Sum	Minimum	Median	Maximum
Variant 1 <0.2 mm	Length	12	20.60714	8.13771	288.5	9.4	20.95	37.3
	Width	12	9.41429	3.27317	131.8	4.1	9.15	16.2
Variant 2 0.2–0.4 mm	Length	7	40.05714	18.09741	280.4	15.7	37.2	58.5
	Width	7	14.47143	5.07599	101.3	8.4	14.5	23.7
Variant 3 0.24–0.6 mm	Length	4	28.45	17.44754	113.8	14.9	22.55	53.8
	Width	5	15.68	8.68976	78.4	8.7	13.7	30.7
Variant 4 0.6–0.8 mm	Length	3	21.66667	17.26306	65	11.6	11.8	41.6
	Width	10	10.14	4.88676	101.4	3.5	10.95	16.4

N is the number of “measuring points”, i.e., places where length and width of the discontinuities were measured.

**Table 5:** The weight percentages of carbon and oxygen in the extruded and non-extruded PLA.

Material	Element	Average [wt.%]	Std. Deviation [wt.%]
PLA before extrusion	C	39.56 ^A^	1.576
	O	60.44 ^a^	
PLA after extrusion	C	39.11 ^A^	3.021
	O	60.89 ^a^	

C—Carbon, O—oxygen, ^A,a^—homogenous groups.

## Data Availability

The original contributions presented in this study are included in the article. Further inquiries can be directed to the corresponding author.
